# Detection of Bovine IgG Isotypes in a PPA-ELISA for Johne's Disease Diagnosis in Infected Herds

**DOI:** 10.1155/2012/145318

**Published:** 2012-06-26

**Authors:** Bárbara Fernández, Liliana Rosa Gilardoni, Ana Jolly, Silvia Beatriz Colavecchia, Fernando Alberto Paolicchi, Silvia Leonor Mundo

**Affiliations:** ^1^Cátedra de Inmunología, Facultad de Ciencias Veterinarias, Universidad de Buenos Aires (UBA), Chorroarín 280, Ciudad Autónoma de Buenos Aires C1427CWO, Argentina; ^2^Laboratorio de Bacteriología, Estación Experimental Agropecuaria Balcarce, Instituto Nacional de Tecnología Agropecuaria (INTA), Ruta 226, Km 73.5, Provincia de Buenos Aires, Balcarce B7620BEN, Argentina; ^3^Departamento de Producción Animal, Facultad de Ciencias Agrarias, Universidad Nacional de Mar del Plata (UNMdP), Ruta 226, Km 73.5, Provincia de Buenos Aires, Balcarce B7620BEN, Argentina

## Abstract

Johne's Disease or Paratuberculosis is a chronic granulomatous enteritis disease affecting ruminants. Detection of subclinically infected animals is difficult, hampering the control of this disease. The aim of this work was to evaluate the performance of detection of IgG isotypes in a PPA-ELISA to improve the recognition of cattle naturally infected with *Map* in different stages. A total of 108 animals from Tuberculosis-free herds were grouped as follows: exposed (*n* = 30), subclinically infected (*n* = 26), clinically infected (*n* = 14), and healthy controls (*n* = 38). Receiver-operating characteristic (ROC) curves of isotypes/PPA-ELISAs were constructed and areas under the curves were compared to evaluate the performance of each test. Our study demonstrated that the conventional PPA-ELISA (detecting IgG) is the best to identify clinically infected animals with high sensitivity (92.9%) and specificity (100%). Meanwhile, IgG2/PPA-ELISA improved the number of subclinically infected cattle detected as compared with conventional IgG/PPA-ELISA (53.8 versus 23.1%). In addition, it had the maximum sensitivity (65.0%, taking into account all *Map*-infected cattle). In conclusion, the combination of IgG and IgG2/PPA-ELISAs may improve the identification of *Map*-infected cattle in different stages of disease. The usefulness of IgG2 detection in serological tests for Johne's Disease diagnosis should be further evaluated.

## 1. Introduction

Johne's Disease (JD) or Paratuberculosis is a chronic granulomatous enteritis disease affecting ruminants [1, 2]. It is caused by *Mycobacterium avium* subsp. *paratuberculosis* (*Map*) and leads to major economic losses in the dairy industry worldwide [3]. *Map* has been implicated as a possible cause of Crohn's disease, which is a chronic granulomatous ileocolitis in humans. However, its role in this pathology remains controversial [4–6].

Calves are the most susceptible category during the first months of life and become infected through ingestion of *Map*-contaminated colostrum, milk, or feces [2, 7]. Fetal transmission is also possible when dams are infected with *Map* [7, 8]. 

During initial infection, the immune response is predominated by a cell-mediated immune profile (Th1). Subclinically infected animals are generally low *Map* fecal shedders and have undetectable levels of *Map*-specific serum antibodies and increasing specific gamma interferon (IFN-*γ*) responses [9]. After a long incubation period (years), a proportion of infected animals develop to a clinical stage, which is characterized by chronic diarrhea, protein-losing enteropathy, cachexia, and eventual death. In addition, increases in bacterial shedding in feces and serum antibody titers have been described in this stage of JD, suggesting a shift of the immune response to a humoral profile (Th2) [1, 10, 11]. The humoral immune response against mycobacterial infections has been considered nonprotective [1, 2]. However, it has been demonstrated that antibodies have an active role in *Map* infection *in vitro*. *Map* immune sera or purified specific antibodies enhance bacterial interaction with macrophages, improve the activation of the nuclear factor NF-kB in infected cells, and affect *Map* intracellular viability [12–14].

The control of JD has been difficult for several reasons. Fecal culture on conventional solid media is expensive, laborious and slow (requiring 6 months for assay ending), and has low sensitivity [15–17]. Detection of cellular immune response by either the skin test or IFN-*γ* production is useful for early diagnosis of infection, but these assays have high variability and low specificity [18, 19]. Vaccines have been demonstrated to decrease the amount of *Map* shedding, to prevent the development of the clinical stage and to reduce the impact on milk production. However, they do not prevent infection and shedding of the bacteria and interfere with Tuberculosis and JD diagnosis [20].

Although conventional ELISA (detecting IgG) has low sensitivity during the subclinical stage of the infection, it is the test most used for JD control due to its low-cost, high-throughput, standardized protocols, and correlation with *Map* fecal shedding levels [21–23]. Various antigens of *Map* have been studied, including protoplasmic antigen (PPA), lipoarabinomannan (LAM), p34 protein carboxy-terminal (P34-cx), purified protein derivative (PPDp), and heat shock proteins (Hsp), of which PPA is the one most used for diagnosis [21–23]. Production of *Map*-specific isotypes switches during the course of the disease [10, 24, 25] with Th1 responses being related to IgM and IgG2, and Th2 responses being related to IgG1 and IgA in cattle [26]. In the same way, high levels of specific IgG1 against several antigens have been detected in sera from *Map*-infected cattle at a clinical stage of the disease [13, 14, 24, 25]. In a previous study, we have shown increases in the levels of *Map*-specific IgG2 in cattle at both the subclinical and clinical stages of JD [25].

The aim of this work was to evaluate the performance of detection of IgG isotypes in a PPA-ELISA to improve the recognition of cattle naturally infected with *Map* in different stages of the disease.

## 2. Materials and Methods

### 2.1. Animals

Sera from 108 Holstein-Frisian bovines from Tuberculosis-free accredited dairy herds from the Pampas region of Argentina were used to assess the performance of IgG, IgG1, and IgG2/PPA-ELISAs.

JD diagnosis was achieved as previously described [25]. Briefly, we examined animals for clinical signs of disease and for *Map* presence in milk and fecal-isolated colonies by PCR identification of the IS*900 *fragment. Milk samples were concentrated by *Map*-specific immunomagnetic beads (NEB, New England Biolabs, Ipswich, MA, USA) [27, 28]. Fecal cultures were carried out in Herrold egg yolk medium with mycobactin J (Allied Monitor Inc., Fayette, MO, USA) and pyruvate (Sigma-Aldrich Corp., St. Louis, MO, USA).

Animals were grouped as follows:

- exposed (E,* n *= 30): from *Map*-infected herds, without clinical signs of JD and negative to IS*900*-PCR (from feces and milk);
- subclinically infected (SC,* n *= 26): from *Map*-infected herds, without clinical signs of JD and positive to IS*900*-PCR (from feces, milk, or both);
- clinically infected (C,* n *= 14): from *Map*-infected herds with chronic diarrhea and positive to IS*900*-PCR (from feces, milk, or both);
- healthy control (Hc,* n *= 38): from *Map*-free herds and negative to IS*900*-PCR (from feces and milk).

### 2.2. ELISAs

IgG, IgG1, and IgG2/PPA-ELISAs were evaluated using sera from the 108 bovines. Cross-reactive antibodies were preadsorbed with *Mycobacterium phlei *[29], which had been grown at 37°C in Middlebrook 7H9 broth (DifcoTM, BD biosciences, Franklin Lakes, NJ, and USA) containing 10% albumin-dextrose-sodium chloride and then heat-inactivated at 85°C for 30 minutes. For preadsorption, sera diluted 1:5 with PBS containing heat-inactivated *Mycobacterium phlei* (optical density (OD) at 600 nm of 1) were incubated at 37°C for 1 h with shaking, and then at 4°C for 16 h. 

Flat-bottomed 96-well polystyrene plates were coated (4°C, 16 h) with 2 *μ*g/well of PPA (Allied Monitor Inc.) in 50 *μ*L of 0.05 M sodium carbonate buffer pH 9.6. The plates were washed three times with rinsing buffer (0.05% Tween 20 in PBS) and blocked with 10% skimmed milk in PBS. All subsequent incubations were performed at 37°C for 1 h and after each incubation, plates were washed three times with rinsing buffer. A volume of 50 *μ*L of preadsorbed sera at a final dilution of 1:5 (for IgG2 analyses) or 1:100 (for IgG and IgG1 analyses) in 5% skimmed milk in PBS was added. The antibodies used were: HRP-conjugated goat anti-bovine IgG (KPL, Kirkegaard & Perry Laboratories Inc., Gaithsburg, MD, USA), HRP-conjugated sheep anti-bovine IgG1 (Bethyl Laboratories Inc., Montgomery, TX, USA), and mouse monoclonal anti-bovine IgG2 (Sigma-Aldrich Co.) followed by HRP-conjugated goat anti-mouse IgG (KPL). Plates were developed using ortho-phenylendiamine dihydrochloride (OPD, Sigma-Aldrich Co.) in citrate buffer (Sigma-Aldrich Co.) and read in an OpsysMR spectrophotometer (Dynex Technologies, Chantilly, VA, USA). Results are expressed as mean OD values at 490 nm.

### 2.3. Data Analysis

All experiments were conducted in duplicate or triplicate and repeated at least twice.

STATISTIX 8.0 (Analytical software, Tallahassee, USA) was used to analyze data of the humoral immune response against PPA. The logarithms of the mean OD values obtained were compared between groups. The levels of IgG and IgG2 were studied with ANOVA followed by Tukey's test, whereas those of IgG1 were analyzed with the Kruskal-Wallis test followed by pairwise comparisons.

CurvMedCalc Software version 12 (Mariakerke, Belgium) was used to evaluate the power of the IgG, IgG1, and IgG2/PPA-ELISAs and to build the Receiver-operating characteristic (ROC) curves of infected cattle. The sensitivity of each test was estimated as % of infected cattle (subclinically infected, clinically infected, or both) testing positive at the cut-off chosen. The specificity of each test was calculated as % of cattle from the healthy control group testing negative at the cut-off chosen. ROC curves for IgG, IgG1 and IgG2/PPA-ELISAs of subclinically and clinically infected cattle were constructed as plots of sensitivity versus 100 minus specificity for each possible cut-off [30, 31]. Different methods were applied to assess the cut-off points of each ELISA. The mean OD values of healthy control group ±2 standard deviation and the ROC curves of infected cattle were analyzed. Cut-off points were selected from the ROC curves in order to obtain the highest sensitivity for subclinically infected cattle with a specificity of at least 90%. The area under each ROC curve (AUC) was estimated and AUCs were compared using the method described by DeLong et al [32].

The level of significance was set at a *P *value < 0.05.

## 3. Results

**Figure 1:**
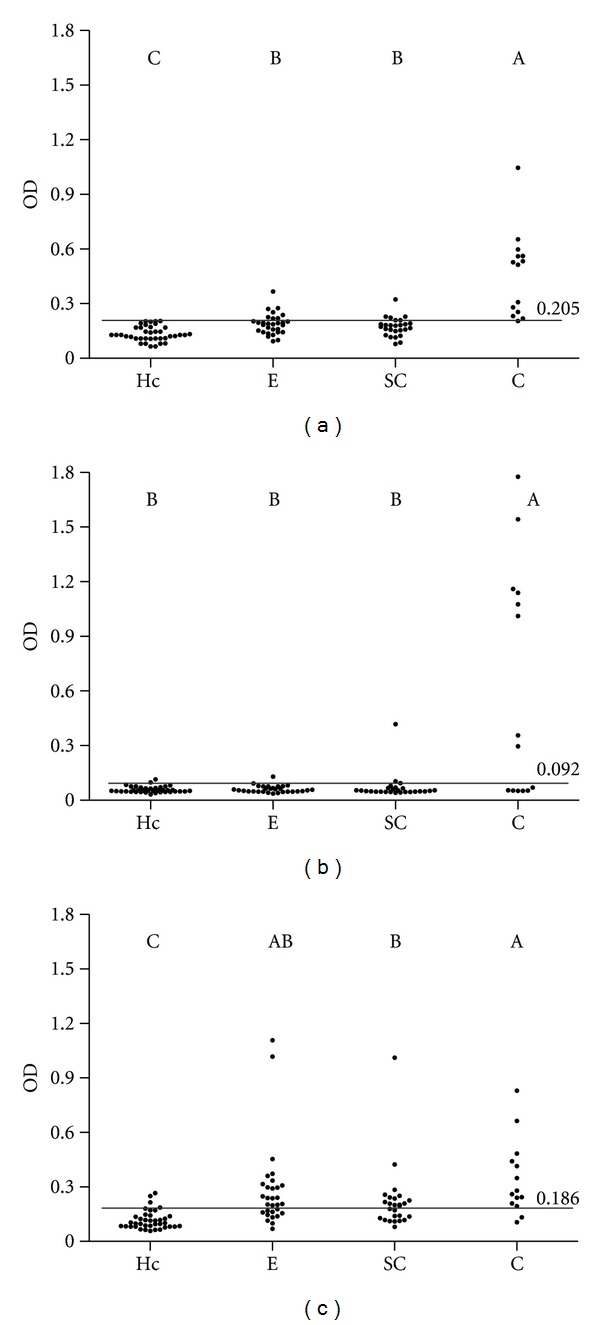
Results of isotypes/PPA-ELISAs. Dotplots of IgG (a), IgG1 (b), and IgG2 (c) PPA-ELISAs. Antibody responses are plotted as mean optical density (OD); lines and numbers (over lines) represent cut-off points. Letters indicate a significant difference (*P*< 0.05) between groups. Groups: healthy controls (Hc, *n* = 38), exposed (E, *n*= 30), subclinically infected (SC, *n* = 26), and clinically infected (C, *n* = 14).

Results of the isotypes/PPA-ELISAs in sera from healthy control, exposed, subclinically infected, and clinically infected cattle are shown in Figure 1 and Table 1. PPA-specific IgG was significantly increased in sera from all groups of *Map*-infected herds (exposed, subclinically infected, and clinically infected) compared with the healthy control group. In addition, the clinically infected group showed the highest values detected. When the groups were evaluated by the IgG1/PPA-ELISA, only the clinically infected group showed high levels of this isotype. Meanwhile, the levels of specific IgG2 were significantly increased in all groups from *Map*-infected herds (*P* < 0.05).

**Figure 2:**
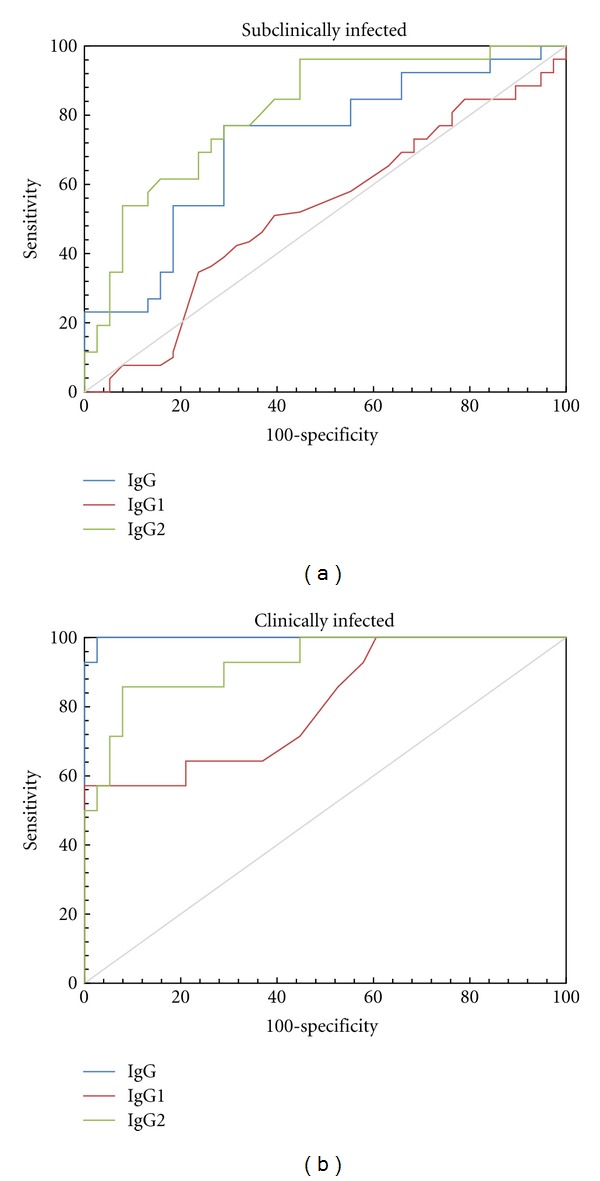
Performances of isotypes/PPA-ELISAs for subclinically infected (a) and clinically infected cattle (b). Receiver-operating characteristic (ROC) curves for IgG, IgG1, and IgG2/PPA-ELISAs.

The ROC curves of the IgG, IgG1, and IgG2/PPA-ELISAs for the subclinically and clinically infected groups are shown in Figure 2. As expected, the AUCs were higher for the clinically infected group than for the subclinically infected one (Table 2). 

The IgG/PPA-ELISA showed the highest specificity (100%) and sensitivity for clinically infected cattle (92.9%, Table 3). However, this test detected as positive only 6/26 of the subclinically infected animals and 8/30 of the exposed animals (Table 1).

The IgG1/PPA-ELISA demonstrated low performance and low sensitivity (27.5% of *Map*-infected cattle (subclinically and clinically infected), Figure 2, Tables 2 and 3).

The IgG2/PPA-ELISA showed 92.1% of specificity and the best performance for the subclinically infected group (AUC = 0.812) as compared with the IgG/PPA-ELISA (AUC = 0.719) and IgG1/PPA-ELISA (AUC = 0.526), detecting 53.8% of the subclinically infected animals and 63.3% of the exposed animals (Figure 2 and Tables 1–3). In addition, the IgG2/PPA-ELISA had the maximum sensitivity (65.0%, taking into account all *Map*-infected cattle) and was able to detect 26/40 of *Map*-infected cattle. In contrast, only 19/40 were identified by the IgG/PPA-ELISA.

## 4. Discussion

The response of isotypes in *Map*-infected cattle has been previously studied [10, 13, 14, 24, 25]. We have described *Map*-specific isotypes detecting high levels of IgG2 in sera from* Map*-infected cattle at both the subclinical and clinical stages of the disease [13, 25]. Taking into account that PPA is the *Map* antigen most widely used [13, 22, 33], in the present work, we developed isotypes/PPA-ELISAs to evaluate their application in diagnosis of JD in cattle. 

It has been described that *Map*-infected animals in the clinical stage are high shedders of bacteria in feces, and thus have the greatest potential to transmit *Map *to other animals of the herd [7, 34]. Meanwhile, subclinically infected cattle usually shed lower levels of *Map* and they are the largest part of the *Map*-infected herds, so detection of these animals is considered of great importance for JD control [35].

In this work, we detected an increase in the level of PPA-specific IgG in sera from clinically infected animals. Similar responses against other *Map* antigens have been previously reported [10, 13, 25]. We also detected increases in the levels of specific IgG in the subclinically infected group, in contrast to our previous study using *Map*-whole bacteria as antigen [25]. The IgG/PPA-ELISA demonstrated a perfect specificity (100%); this is in accordance with published studies that have described specificities from 94 to 100% [21, 36].

Although specific IgG1 against *Map*-antigens has been described as characteristic of clinically infected animals [13, 14, 24, 25], in the present study the detection of PPA-specific IgG1 did not improve the diagnosis in this stage of disease. 

Interestingly, the IgG2/PPA-ELISA allowed detecting the majority of subclinically and clinically infected animals, confirming our preliminary studies [13, 25]. 

Although sera were preadsorbed, three animals of the healthy control group showed OD values higher than the cut-off of the IgG2/PPA-ELISA (Figure 1 and Table 1). This could be related to the lower specificity (92.1%). 

Our study demonstrates that the IgG/PPA-ELISA is the best to identify clinically infected animals, with high sensitivity and specificity, in accordance with the accepted statement that conventional ELISAs mostly identify this category of infected cattle [21, 23]. 

On the other hand, our IgG2/PPA-ELISA improved the number of subclinically infected cattle detected as compared with conventional IgG/PPA-ELISA (53.8 versus 23.1%), maintaining high levels of specificity. Nevertheless, this sensitivity is slightly lower than that reported by Paolicchi [33] using an IgG/PPA-ELISA, although this could be related to the number of animals included (26 versus 8 animals). 

The sensitivity of fecal culture has been reported to be too low to define absence of *Map *infection for animals residing in known infected herds [21]. In fact, *Map*-infected cattle in the early stage may shed bacteria under detectable levels using current methods, including culture and PCR [35]. In the same way, Nielsen [37] has recently highlighted the importance of the study of *Map*-infected shedder and nonshedder animals to evaluate an immune-based diagnostic test. Thus, in the present work, we incorporated a group of exposed animals from *Map*-infected herds, excluding them from the specificity and sensitivity analysis. In this group, the use of IgG2/PPA-ELISA allowed detection of more positive animals than the other isotypes evaluated (63.3 versus 26.7 or 3.3%). Using a IgG/PPA-ELISA test, Huda et al. detected 11% of exposed animals as positive [31]. 

New antigens have been proposed to increase the sensitivity of JD diagnosis by IgG/ELISA [34, 36, 38]. Thus, it could be interesting to evaluate those antigens in an IgG2/ELISA.

In conclusion, our results show that IgG2/PPA-ELISA improves detection of subclinically *Map*-infected cattle or herds with animals in all stages of JD and in combination with IgG/PPA-ELISA may improve differentiation of clinical stages of disease. More studies should be conducted to better approach the utility of the IgG2/PPA-ELISA, in which, naturally and experimentally infected cattle should be included and the infection status should be supported by histopathological examination and culture of tissues. In addition, the usefulness of IgG2 detection in serological tests for Johne's Disease diagnosis should be further evaluated.

## Figures and Tables

**Table 1:** Percentages of positivity of isotypes/PPA-ELISAs.

Groups	IgG/PPA-ELISA	IgG1/PPA-ELISA	IgG2/PPA-ELISA
Hc	0.0% (0/38)	5.3% (2/38)	7.9% (3/38)
E	26.7% (8/30)	3.3% (1/30)	63.3% (19/30)
SC	23.1% (6/26)	11.5% (3/26)	53.8% (14/26)
C	92.9% (13/14)	57.1% (8/14)	85.7% (12/14)

Numbers of positive animals are shown between brackets. Groups: healthy controls (Hc, *n* = 38), exposed (E, *n* = 30), subclinically infected (SC, *n* = 26), and clinically infected (C, *n* = 14).

**Table 2:** Performances of isotypes/PPA-ELISAs for subclinically infected and clinically infected cattle.

Subclinically infected				Clinically infected			
PPA	AUCs	Comparison of AUCs		PPA	AUCs	Comparison of AUCs	
ELISA				ELISA			
IgG	0.719	IgG ~ IgG1	*P* = 0.0858	IgG	0.998	IgG ~ IgG1	*P* = 0.0057
IgG1	0.526	IgG ~ IgG2	*P* = 0.2402	IgG1	0.805	IgG ~ IgG2	*P* = 0.0721
IgG2	0.812	IgG1 ~ IgG2	*P* = 0.0048	IgG2	0.927	IgG1 ~ IgG2	*P* = 0.0913

Estimated area under the curve (AUC) of each test and pairwise statistical analysis.

**Table 3:** Specificity and sensitivity of isotypes/PPA-ELISAs.

PPA	Specificity	Sensitivity		
ELISA		Subclinically	Clinically	Total
IgG	100.0%	23.1%	92.9%	47.5%
IgG1	94.7%	11.5%	57.1%	27.5%
IgG2	92.1%	53.8%	85.7%	65.0%

The specificity of isotypes/PPA-ELISAs was calculated as % of cattle from the healthy control group testing negative. The sensitivity of each test was estimated as % of infected cattle (subclinically infected, clinically infected, or both) testing positive.
